# Colloidal Liquid Crystals Confined to Synthetic Tactoids

**DOI:** 10.1038/s41598-019-56729-9

**Published:** 2019-12-31

**Authors:** Ioana C. Gârlea, Oliver Dammone, José Alvarado, Valerie Notenboom, Yunfei Jia, Gijsje H. Koenderink, Dirk G. A. L. Aarts, M. Paul Lettinga, Bela M. Mulder

**Affiliations:** 1 AMOLF, Department of Living Matter, Amsterdam, 1098XG The Netherlands; 2 University of Vienna, Faculty of Physics, Vienna, A-1090 Austria; 3 Kavli Institute of Nanoscience, Delft University of Technology, Department of Bionanoscience, Delft, 2629HZ The Netherlands; 4 University of Oxford, Department of Chemistry, Oxford, OX1 3QZ UK; 5 Forschungszentrum Jülich, Institute of Complex Systems (ICS-3), Jülich, 52425 Germany; 6 KU Leuven, Laboratory for Soft Matter and Biophysics, Leuven, B-300 Belgium

**Keywords:** Colloids, Liquid crystals

## Abstract

When a liquid crystal forming particles are confined to a spatial volume with dimensions comparable to that of their own size, they face a complex trade-off between their global tendency to align and the local constraints imposed by the boundary conditions. This interplay may lead to a non-trivial orientational patterns that strongly depend on the geometry of the confining volume. This novel regime of liquid crystalline behavior can be probed with colloidal particles that are macro-aggregates of biomolecules. Here we study director fields of filamentous *fd*-viruses in quasi-2D lens-shaped chambers that mimic the shape of tactoids, the nematic droplets that form during isotropic-nematic phase separation. By varying the size and aspect ratio of the chambers we force these particles into confinements that vary from circular to extremely spindle-like shapes and observe the director field using fluorescence microscopy. In the resulting phase diagram, next to configurations predicted earlier for 3D tactoids, we find a number of novel configurations. Using Monte Carlo Simulations, we show that these novel states are metastable, yet long-lived. Their multiplicity can be explained by the co-existence of multiple dynamic relaxation pathways leading to the final stable states.

## Introduction

The local structure of materials can be strongly influenced by confinement, either when this confinement is externally imposed on the material by bounding surfaces, or whenever localized interfaces between different states of the same material arise spontaneously e.g. through a micro-phase separation process. In this paper we discuss the typical example of lyotropic liquid crystals (LC). The droplets that form during the isotropic-nematic (I-N) phase separation of lyotropic liquid crystals often have a peculiar tactoidal shape, which was first observed in the early 20th century in a synthetic system of vanadium pentoxide particles (1925)^1^, and later in a biological system of Tobacco Mosaic Virus particles (1936)^2^. This shape is set by the competition between elastic properties of the nematic LC phase, the interfacial tension and the anchoring condition at the nematic to isotropic interface^3,4^. As droplets are small at the onset of phase separation, they have a high aspect ratio due to the elasticity. The aspect ratio decreases as the so-called ‘tactoids’ grow, due to the interfacial tension. It is predicted that this evolution of shape is accompanied by a transition from a homogeneous director-field, where all director lines are parallel to the main axis of the shape, to a bipolar structure, which is characterized by two point defects at the cusps and director lines that follow the contour of the interface^3–6^. The latter structure is favorable, because, for entropic reasons, colloidal rods preferably align parallel to walls^7^. Thus, the shape of the droplet depends on the time elapsed after the onset of the phase separation, as was observed for several rod-like viruses^3,8^. As a consequence, the structure of the director field can only be studied for a fixed set of shapes that evolve along a predetermined path in time as they grow. Remarkably, the mitotic spindle is also an assembly of microtubules with a tactoidal shape. Its construction involves a host of active components, and it is not simply an equilibrium system. Nevertheless, recent work has shown that it is well described as a confined (active) liquid crystal system^9^. Also in this case, the shape is determined by the outcome of the internal competition between active stresses and surface tension, and hence difficult to control independently.

A systematic approach to studying the structure of a liquid crystalline director field in confined geometries is provided by soft lithography. This technique enables essentially arbitrarily shaped micron-sized planar chambers to be made, for example to mimic the organization of F-actin in a cell^10^. Recently, this approach was used to tune the structure of the director field of a dispersion of filamentous *fd* virus particles in the nematic phase confined to wedge-shaped cells^11^. The *fd* virus can be considered as an almost ideal rigid rod, with an aspect ratio of 120 and a persistence length of more than twice its contour length of 880 *nm*^12^. Among others, a bend-to-splay transition was observed with increasing opening angle of the wedge. Using simulations it was also shown that this transition depends on the stiffness of the rods, which controls the ratio of the bend and splay elastic constants. The experimental beauty of this type of work is that these mesogens are so large that fluorescence microscopy can be used to directly obtain the director field. At the same time their length is not much smaller than the length scales accessible with soft lithography. This means that they can be used to probe the physics of confined liquid crystals at the scale where the size of the particles themselves also becomes a relevant variable. This feature was recently exploited to identify a break down of continuum theory both in rectangular and donut-shaped micron-sized chambers^13–15^.

**Figure 1:**
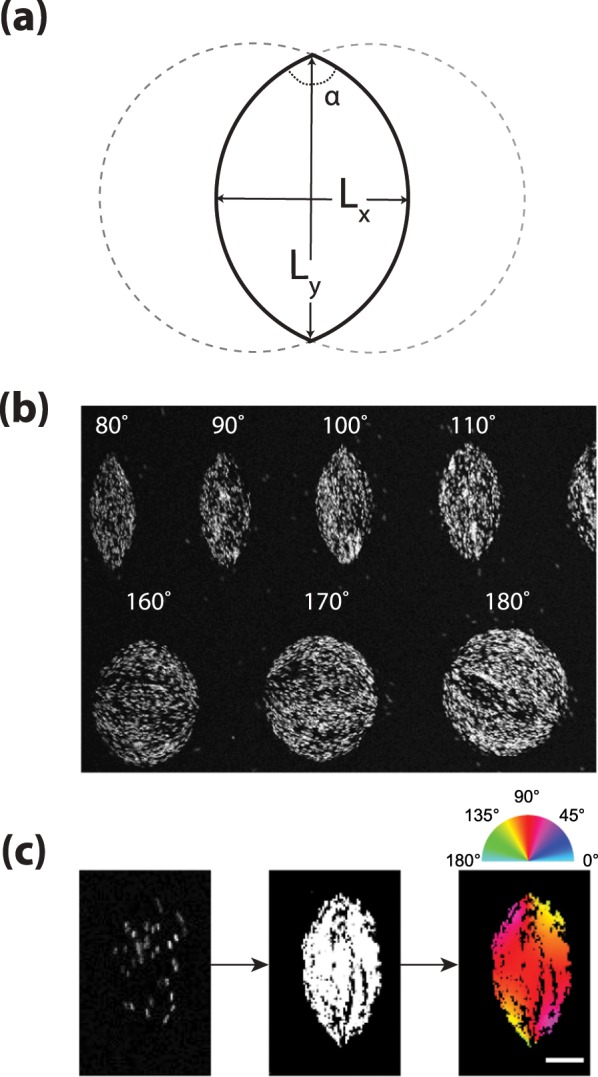
(**a**) Schematic of the tactoid geometry. (**b**) Subset of the experimentally employed wells organized according to their opening angle *α* (*L*_*y*_ = 30*μ*m, maximum intensity projection image). (**c**) From left to right: Example of experimental image (single frame), maximum intensity projection of all frames collected for the same well, and image obtained after orientational analysis (scale bar 5*μm*).

Here, we confine nematic dispersions of *fd* virus particles to shallow, quasi two-dimensional lens-shaped chambers. Thus we mimic the uncontrolled complex non-equilibrium 3D confinement problem of a I-N phase separating system by a fully controllable 2D confinement of which we can *independently* vary the aspect ratio *L*_*y*_/*L*_*x*_ as well as the overall system size, given by the dimension of the long axis *L*_*y*_ (see Fig. 1). We combine the experiments with Monte Carlo simulations, in order to gain further insight in the evolution of the structures, which cannot be experimentally accessed. This combined experimental and *in silico* approach allows us to study the fully equilibrated structure of the nematic for fixed sizes and aspect ratios. Moreover, we can investigate the influence of the finite particle size, which is neglected in the continuum description^3–5^ and difficult to access for typically nanometer-sized thermo-tropic liquid crystals.

Experiments and simulations on hard rods in small circular confinement, which is one of our extreme cases, have been performed previously. Monte Carlo simulations at moderate^16^ and high concentrations^17^ already clearly show finite size effects and a transition to a bipolar structure for sufficiently big cavities. Interestingly, experiments on vibrofluidized granular rods, where finite size effects should play a role, could be interpreted with continuum theory and a transition between a bipolar and homogeneous director field has been observed. However, it is unclear in what sense these systems can be considered to really be in thermal equilibrium, as fluctuations were high and non-polar defects were observed^18^. This points to the importance of establishing whether a system has equilibrated or not. Homogeneous configurations have also been observed for tactoids of carbon nano-tubes^19^, which are particles with extremely high stiffness, while tactoids of colloidal rods like *fd*-viruses^20–22^ and F-actin^23–25^ are bipolar, as are tactoids of liquid crystals of non-biological origin^26–30^. Recent experimental work on amyloid fibril cholesteric tactoids shows a homogeneous to bipolar transition as function of the volume of the tactoid^31^. This confirms the theoretical predictions that this transition from a bipolar to a homogeneous configuration is determined both by the stiffness of the particles^5,6^ and the aspect ratio^4,32^.

Here, we will show that transitions between different states can also be obtained by tuning the size and shape of the confining volume of the nematic *fd*-dispersions. As the layers we employ are very thin ($\lesssim 3\,\mu {\rm{m}}$) we use fluorescence microscopy to obtain the director field instead of the generally used and more coarse-grained polarization microscopy. Monte Carlo simulations are then performed in order to test if the experimentally obtained director fields are the stable equilibrium patterns or depend on initial configurations.

## Results

### The diagram of states: Identification of director-fields

**Figure 2:**
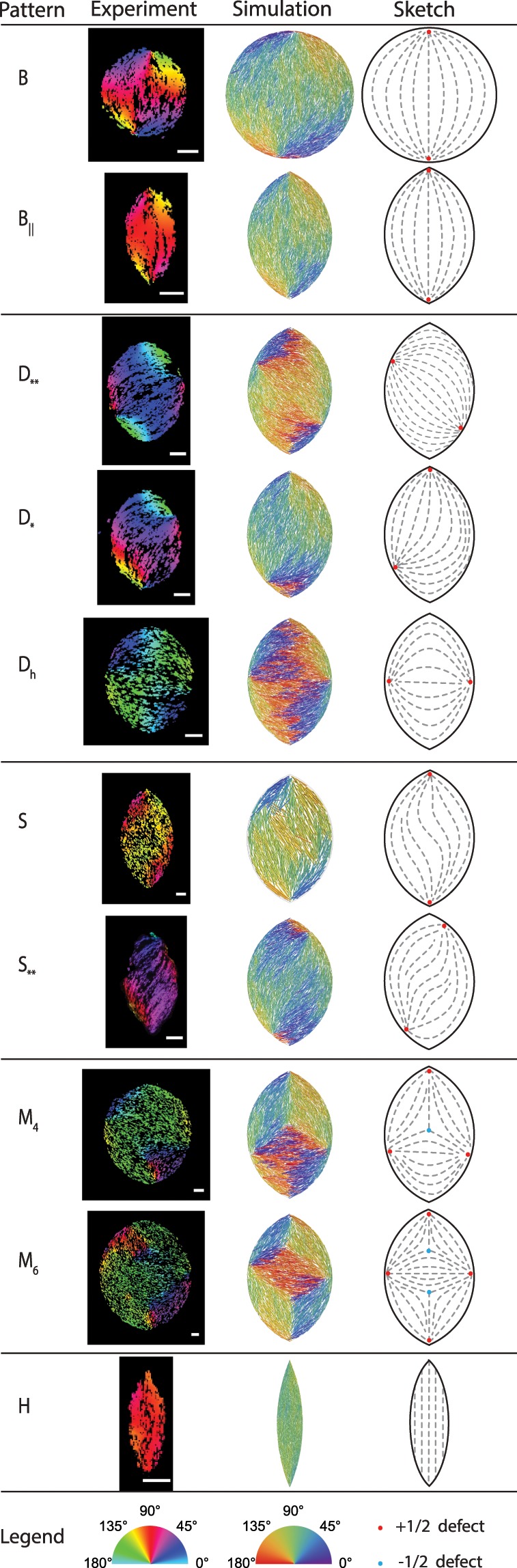
Overview of the different director structures as obtained from experiments and simulations. The right most column shows a sketch of the nematic director (dashed gray lines) where the defects points are marked in color according to their topological charge (red for the $+\frac{1}{2}$ singularities and blue for the $-\frac{1}{2}$). For all experimental images the scale bar is 5 *μm*. Simulation parameters: *L*_*y*_/*L*_*x*_ = 1.5 everywhere, except for *B* where *L*_*y*_/*L*_*x*_ = 1 and *H* where *L*_*y*_/*L*_*x*_ = 5; *Ly* = 300*d* for *B*, *B*_∥_, D_**_, *D*_*h*_, and S_**_, *Ly* = 270*d* for D_*_ and *M*_4_, *Ly* = 150*d* for *S*, *Ly* = 240*d* for *M*_6_, *Ly* = 400*d* for *H*.

We identified a wide variety of director structures experimentally as well as *in silico*. The pattern of the director-field depends on both the overall size *L*_*y*_ and aspect ratio *L*_*y*_/*L*_*x*_ of the tactoid-shaped confining volume. Figure 2 gives an overview of all configurations that we identified. In the left column we show the color-coded experimental fluorescence images, where the color scale maps to the angle with respect to the short axis of the sample. In the middle column, these patterns are compared to similar structures that we found in the MC simulations, to be discussed in the next section. The rightmost column shows a schematic of the director field corresponding to each pattern. In all, we identified five classes of director field patterns, two of which were already previously predicted by theory^3–5^, namely the bipolar structure *B* and the homogeneous structure *H*. We distinguish between two types of predicted bipolar structures: the one enclosed in a circular domain (denoted by *B*) and the one appearing in a domain with *L*_*y*_/*L*_*x*_ ≠ 1 possessing a unique symmetry axis, where the two defect points that characterize the pattern are located exactly in the cusps of the tactoid (denoted by *B*_∥_). The *H* pattern is characterized by a homogeneous nematic director aligned with the main axis of the tactoid, with no observable defect points. In addition to the two expected classes of patterns we find three additional ones: The first one, *D*, is defined by a bipolar arrangement where one or both defect points are not in the theoretical predicted location of the cusps but located diagonally. We differentiate between structures where only one of the defect points, even if it is positioned on the boundary, is not located in a cusp, D_*_, and where both defects are misplaced, D_**_. The pattern that we denote by *D*_*h*_ is a particular type of D_**_ where the two defects are at opposite locations on the minor axis of the tactoid. The second category of patterns that we observed features a wavy nematic director pattern similar in shape to the letter “S”. The defects can either be located in the cusps, *S*, or outside of the cusps, S_**_. In the *S*-pattern the nematic director can vary significantly over short distances, which in combination with our choice of color-coding the orientations may give the impression that nearby rods are even perpendicular to each other (see upper left quarter of the simulated *S*-pattern in Fig. 2), but this is a visual artefact. Finally, we observe configurations where multiple nematic domains coexist inside the same confining volume, separated by line defects, starting and ending in defect points. We call this type of pattern *M*_*n*_, where *n* is the number of defect points. In Fig. 2 we show two types of *M*-structures, *M*_4_ and *M*_6_. Note that the sum of the topological charge of the defect points for all configurations is equal to 1, matching the Euler characteristic of the container^33,34^.

**Figure 3:**
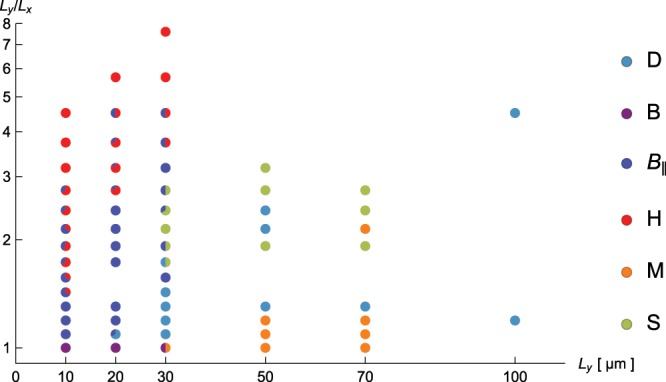
The diagram of states of *fd*-viruses in quasi-2D tactoid-shaped confinement, with system-size given by *L*_*y*_ on the *x*-axis and aspect ratio *L*_*y*_/*L*_*x*_ on the *y*-axis. Patterns are color-coded according to the legend on the right, where for clarity all sub-types of *D*, *S* and *M* patterns were labeled with the same color. Bi-colored points encode the relative frequency of occurrence of the types of patterns corresponding to the colors. For each point in the diagram, we analyzed between 1 and 6 wells.

Having classified all the observed configurations, we can now set up a diagram of states as a function of the major axis *L*_*y*_ and the aspect ratio *L*_*y*_/*L*_*x*_ as shown in Fig. 3. For small system sizes, the trends follow those predicted by Prinsen and van der Schoot^3,4^, with a transition from a *H* structure for high aspect ratio tactoids to a *B*_∥_ or *B* structure for lower aspect ratios. However, for larger system sizes we observe that the *H*-*B* transition shifts to higher aspect ratios, indicating a system size-dependent shift in the trade-off between the cost of aligning with the boundaries versus maintaining the defect points. The novel *S*, *D*, and *M* type structure are mainly found for larger system sizes. The multi-domain pattern *M* seems to be favorable at low aspect ratios, whereas the wavy *S*-type is more prevalent for high aspect ratios. As the occurrence of these director fields patterns does not seem to be energetically favorable, we developed a simulation methodology to test the stability of the experimentally observed patterns, which we discuss in the next section.

### Metastability of phases: equilibration pathways

We implemented standard Monte Carlo simulations of hard spherocylindrical particles of length *L* and diameter *d* confined to quasi 2D tactoid-shaped containers. For technical details of these simulations we refer to the Materials and methods section. With these simulations we can study the evolution of these systems from controlled initial conditions, using the number of MC-steps as a proxy for time.

We first considered the case where all particles are initially aligned to the main axis of the tactoid, i.e. similar to a bulk nematic organization with the nematic director parallel to the long axis *L*_*y*_ or, in other words, the *H* pattern. In this case, we expect only two types of outcome: either the particles equilibrate predominantly only in their center-of-mass degrees of freedom, but without significantly changing the aligned *H* pattern in which they started, or they accommodate to the preferred wall alignment, while at the same time keeping their co-alignment intact as much as possible, in which case the *B*-type pattern emerges. In either case the equilibrium can be reached by minimal rearrangements of particles from their initial configuration. Whether the *B* or the *H* pattern is stable depends on the aspect ratio *L*_*y*_/*L*_*x*_, with the *H* pattern predominant at high aspect ratios. Whether there is indeed a sharp transition between these two states and, if so, where this transition is located is unfortunately hard to assess. This would require a computationally very expensive analysis with high statistics of the local orientational ordering fields to obtain a reliable marker for the transition. We chose not to dwell on this issue here.

**Figure 4:**
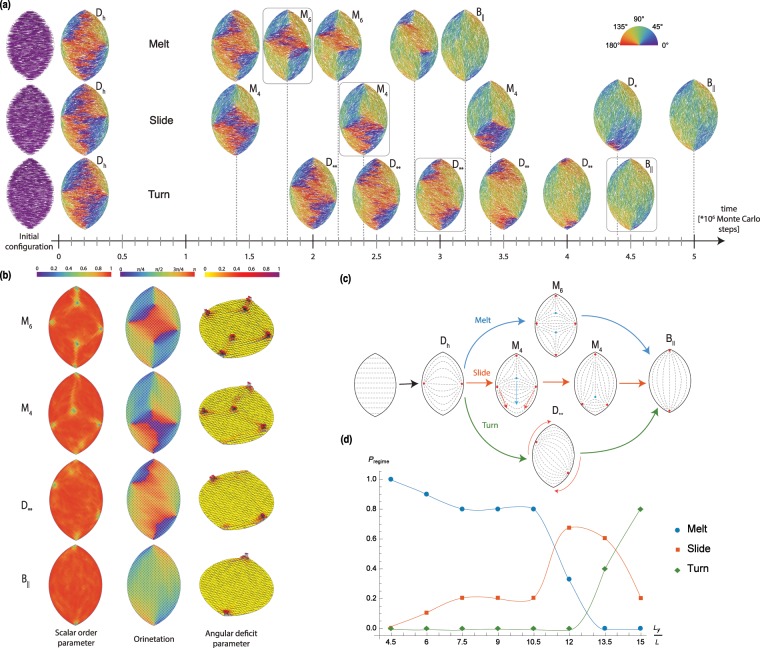
Melting trajectories. (**a**) Snapshots taken along the three equilibration pathways. (**b**) Scalar order parameter, nematic director and disclination parameter for some of the representative states corresponding to the framed configuration in panel (**a**). (**c**) Schematic showing the relocation of defects along the pathways. (**d**) Probability of appearance of the three pathways as a function of the width of the box. Curves are guidelines to the eye. Ten independent runs were performed for each *L*_*y*_/*L*. For all simulations presented in this figure *L*/*d* = 20, *L*_*y*_/*L*_*x*_ = 1.5. For each of the three pathways: *L*_*y*_/*L* = 12 (melt), *L*_*y*_/*L* = 13.5 (slide), and *L*_*y*_/*L* = 15 (turn).

We next turned our attention to a much less favorable starting configuration, in which all particles are initially aligned to the short *L*_*x*_ axis of the tactoid. By starting from this configuration, all particles will need to fully reorient in order to reach either one of the theoretically predicted patterns. We track the evolution of the pattern throughout the duration of the simulation, considering confining containers with aspect ratio *L*_*y*_/*L*_*x*_ = 1.5 but of various sizes (*L*_*y*_/*L* ∈ [4.5, 15]). Irrespective of the tactoid size, we observe, see Fig. 4, that particles initially reorganize relatively fast into a *D*_*h*_ pattern. This typically happens within 5% of the total simulation time needed for reaching the equilibrium configuration. Arguably this pattern can be understood as the most readily accessible configuration that accommodates the two competing thermodynamic driving forces in the system: the tendency towards mutual alignment (already satisfied by our starting configuration) and the preferential alignment of particles to the walls. Surprisingly, we observe that the equilibrium bipolar pattern *B*_∥_ can be reached not by a single sequence of intermediate patterns, but by three distinct equilibration pathways.

The first of these pathways presented in Fig. 4(a) is characterized by the formation of a nematic drop in the center of the container, the nematic director pointing along *L*_*x*_. The droplet is surrounded by 4 other nematic domains each aligned to the average orientation of the part of the wall it is close to. Upon closer inspection of this pattern, we find that the five nematic domains are separated by defect lines, each of them starting in a defect point with topological charge +1/2 located on the boundary of the container and ending pairwise in two defects with charge −1/2 located on the long axis in the inner region (see Fig. 4(b) and the Supplementary Movie S1). The defects can be clearly recognized by the sharp local variation of the orientation of the particles, the co-localized decrease in the scalar order parameter, and the co-occurrence of a maximum of the so-called angular deficit parameter, which we define as the angular variation of the nematic director around a point and is therefore maximal at the defects. For more information on the quantities used to characterize the pattern, please see the Methods section. This *M*_6_ pattern remains stable for a significant amount of time. Ultimately, the nematic droplet in the center becomes unstable at some point during the simulation, after which it is almost instantly disassembled, giving birth to the *B*_∥_ pattern. The droplet appears to melt away, with the two negatively charged defects annihilating the two positively charged ones most adjacent to the central nematic domain. The two remaining defect points are the ones in the cusps which characterize the *B*_∥_ pattern. We will refer to this pathway as “melt”. Sometimes the dissolution of the nematic droplet in the middle of the container results in the formation of a *S*-pattern which has a much shorter life-time than the droplet. This pattern also relaxes to a *B*_∥_ configuration. Since this phenomena does not systematically happen we do not consider the formation of the *S*-pattern as a feature of the “melt” pathway.

The second pathway, which we call “slide”, features the assembly, out of the *D*_*h*_ configuration, of a multi-domain nematic with four defect points: three of them of charge +1/2 and one with charge −1/2, the *M*_4_ pattern. As in the case of the “melt” scenario, the negatively charged defect point is located inside the container whereas the positive ones are on its boundary, one of them already at one of the cusps. All four defects tend to be on one side of the *L*_*x*_-axis, with the pattern on the other side of this axis still resembling the *D*_*h*_ pattern, with only a single bend in the nematic director pattern that starts from one of the defects in the vicinity of the *L*_*x*_-axis and ends in the one opposite while mostly following the wall orientation. Again, the oppositely charged defects are connected by defect lines. In the course of the simulation, the three point defects that are located outside the cusp slowly converge on each other, while collectively “sliding” towards the cusp that contains the bend (see Fig. 4(c) and the Supplementary Movie S2). Due to this synchronous movement and the geometry of the confining volume, the distance between the defects decreases, eventually leading to the annihilation of the −1/2 defect point with one of the +1/2. At the time of this annihilation event the remaining +1/2 point defect is generically not yet in the cusp (i.e. the D_*_ pattern), but in time moves along the boundary until it reaches its equilibrium position in the cusp.

The third equilibration pathway does not feature the formation of additional pairs of point defects, as in the other two cases. The line connecting the two +1/2 point defects, that appear at the beginning of the simulation on the opposite ends of the minor axis, slowly starts rotating, with the whole pattern following this rotation. In this way the system goes through a succession of D_**_ patterns. The initial direction, either clock-wise or counter-clock-wise, of the rotation is arbitrarily chosen. The D_**_ pattern continues to rotate until the two defects reach the cusps and become arrested, thus establishing the *B*_∥_ configuration (see Supplementary Movie S3). Since the pattern effectively rotates into the equilibrium configuration, we call this pathway“turn”.

Each of the equilibration pathways consists of series of patterns that survive for a sufficient time during the simulation to be able to qualify them as metastable, and which also allows us to gather enough statistics to characterize them. The metastability of the patterns is evaluated by its survival longer than it takes for one rod to diffuse over a distance equivalent to the box size (see highlighted rods in the Supplementary Movies S1–S3). We posit that the metastability of these configurations also explains why these patterns are observed in the experiments. Even if other patterns appear along the equilibration pathways (such as the configurations on the “melt” trajectory formed during the disassembly of the central nematic droplet), they are extremely short-lived, which is also consistent with our inability to observe them experimentally.

The propensity for a specific equilibration pathway being chosen by the system depends on the size of the confining container (see Fig. 4(d)). For smaller containers the “melt” scenario constitutes the favored equilibration path, while for bigger containers the “turn” pathway is preferred. The “slide”-type equilibration has significant frequency of occurrence only in the cross-over size regime between the other two pathways. This can be understood by the fact that our system is governed by the competition between wall alignment and particle-particle alignment. The effect of wall alignment extends into the liquid crystal only on the order of length of the rods^7^, so it has a more significant contribution in smaller containers. In these cases the propensity to align strongly to the wall offsets the free energy cost of creating defects. In the bigger containers a smaller fraction of the rods is directly influenced by the wall, and therefore satisfying the particle-particle alignment becomes dominant. In the region where the “slide” pathway appears, the two interactions seem to be equally influential: the intermediate pattern in the “slide” pathway literally appears to be a combination of the one appearing in the other two pathways, with one half of the system organized as half of an *M*_6_ pattern while the other half features a bend characteristic of half of the *D*_*h*_ pattern.

**Figure 5:**
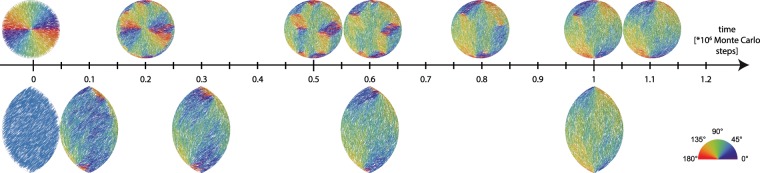
Snapshots taken along the equilibration pathways starting from the hedgehog configuration enclosed in a disk (top) and from an oblique arrangement in a spindle. Simulation parameters for the top row: *L*_*y*_/*L*_*x*_ = 1, *L*_*y*_/*L* = 15 and for the bottom row: *L*_*y*_/*L*_*x*_ = 1.5, *L*_*y*_/*L* = 15.

The only type of pattern that we did not observe so far in the simulations (as a metastable one) is the wavy “S” pattern. Since this type of pattern was observed in the experiments, we assume there must exist an initial arrangement which passes through “S”-type patterns on the way to equilibrium. We therefore tried an initial condition in which all rods point in a direction tilted by 45 degrees with respect to the *L*_*x*_ axis. In this case we find that, as expected, a pattern exhibiting two point defects is assembled. Surprisingly, however, the two defect points appear much closer to the cusps than we would expect from the initial direction of the nematic director, and the rods located in the central region of the container subsequently rotate around the center of the box, with no accompanying movement of the defects, to align their director with the line connecting the two defects. In this way a S_**_ pattern is formed that in the next stage further rotates into the equilibrium configuration (see lower row of Fig. 5).

We now asked the question: what would be the most *unfavorable* initial configuration that we can start with? As the preferred anchoring to the wall is planar, this suggests a configuration where the rods are perpendicular to the wall. Such a configuration is approximately created by letting the rods point radially away from (or equivalently towards) the center of the container. This initial configuration has a single defect of topological charge +1 in the center of the container, also called a “hedgehog” configuration. This arrangement is not only disadvantageous from the perspective of the wall-rod interaction, but also from the rod-rod alignment due to the high degree of splay deformation, and therefore highly unstable. Once we start our simulation the particles in the vicinity of the wall start aligning to it, and various nematic domains appear (see Fig. 5). The central defect splits up into “daughter” defects, thus creating more nematic domains with various orientations. In the course of the simulation, the “daughter” defects move such that the larger nematic domains grow at the expense of smaller ones, until the system finally reaches the equilibrium *B* configuration.

**Figure 6:**
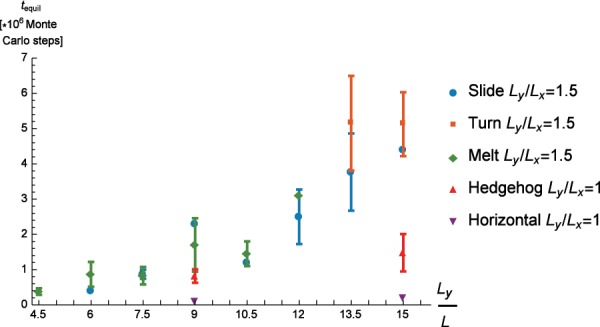
Equilibration time needed as a function of box width for different initial arrangements and geometries of the confinement.

We also studied the equilibration time as a function of the container size for the three canonical equilibration pathways (see Fig. 6). In all three cases, equilibration time increases with the size of the confining container, since in a bigger container the inter-particle co-alignment propagates slower as each rod only tends to align to its immediate surrounding, with long range order taking more time to be established. We see no significant difference in equilibration time between the “melt” and the “slide” pathway. However, the “turn” pathway is consistently slower in equilibrating than the “slide one”. Considering that the “turn” pathway consists of a essentially global rotation, which involves constant rearranging of all the rods, whereas in the “slide” pathway one part of the pattern remains virtually unchanged during the movement of the defects, it is understandable why the former pathway takes longer to equilibrate. For comparison, we also present the equilibration times for a circular geometry with both homogeneous and hedgehog starting configuration. The initially homogeneous configurations equilibrate on a similar time scale that is necessary for the *D*_*h*_ pattern to be assembled in the tactoidal geometries. The hedgehog starting configuration also equilibrates faster in the circular geometry than in the tactoid geometry since, even if in this case the particles need to reorient significantly to reach an equilibrium configuration. Due to circular symmetry all global ordering directions are equivalent and there is no need for the nematic director to align along a specific major axis in order to reduce bending.

## Discussion

Our microfabricated chambers allowed us to independently explore the effects of shape and overall size of tactoidal liquid-crystalline domains. For the smaller system sizes (*L*_*y*_ ≤ 20 *μm*) the previously predicted bipolar (*B*_∥_) and homogeneously aligned (*H*) equilibrium states are recovered. Although we could establish that the latter state is predominant at high aspect ratios, pinpointing the phase boundary between the two states remains a challenge, also for simulations. For larger system sizes (*L*_*y*_ ≥ 30 *μm*) an additional number of novel states are identified. Our Monte-Carlo simulations indicated that these novel states are meta-stable but increasingly long-lived as the system size increases. Their relaxation towards the equilibrium state depends on essentially global rearrangements of the patterns, either through annihilation of defects or a global rotation of the preferred axis. A striking observation from the simulations is that multiple independent relaxation pathways may occur whose probability depends on the shape and size of the domain. The observation of the meta-stable patterns in experiments (instead of the expected equilibrium bipolar pattern) could also be the consequence of the individual characteristics of the wells such as the surface roughness of the walls. This could lead to the slowing down or even arrest of the pattern, for example along the “turn” pathway, by anchoring the rods to the walls.

It is apposite to ask to what extent some of the results we have obtained are specific to the tactoidal shape. Indeed, in our earlier work on square^35^, rectangular^13^ and circular geometries^15^ we did not observe any long-lived meta-stable states. In the square and rectangular case, the ordering is fully dominated by the plane boundaries and multiple corners, leading to immediate orientational “lock-in” of the final patterns, while in the circular case, the symmetry removes any driving force towards a specific final orientation. It appears, that the tactoidal shape is therefore in a sense an interesting intermediate case: on the one hand the twin antipodal cusps provide enough of a cue to fix the ultimate overall orientation axis, while on the other hand the smooth boundaries allow intermediate states, which can only slowly relax to exist for longer times. This suggests that, apart from their intrinsic interest, our results have implications for the design of micron-scale wells containing colloidal LCs. Ideally one would like robust control over the final configuration, and clearly long-lived intermediate structures are potential impediment towards this goal. This raises the interesting question whether the boundary of the domain can be designed in such a way that the metastable states are optimally suppressed, or, conversely, that certain non-trivial defect-mediated domain structures can be stabilized.

The wavy *S*-patterns observed may at first appear similar to the the twisted tactoidal phases described by R. D. Williams^36^. However, we believe that they are unrelated. The conditions for the appearance of the twisted phase following Williams reads^36^: *K*_11_ ≥ *K*_22_ + 0.431*K*_33_, where *K*_11_, *K*_22_, and *K*_33_ are the Frank elastic constants related to splay, twist and bend, respectively. For our experimental system, the splay and bend constants are about equal (*K*_11_ ≈ *K*_33_ ≈ 10^−8^*dyne*)^11,37^, and the measured value for the twist constant is *K*_22_ ≈ 10^−7^*dyne*^38^. Considering these values, the condition for the appearance of the twisted phase are definitely not met as the right-hand side term of the inequality is an order of magnitude larger than the one of the left hand side, so one would expect that (for 3D tactoids) only the tangential bipolar arrangement is observed. Furthermore, while we cannot completely exclude that a twist in the perpendicular plane to our patterns exists, we think that this would be highly unlikely to affect our patterns. The cholesteric pitch for our system would be around 100 *μm*^38^, whereas our chambers have a depth of only 3 *μm*, i.e. too small for the twist to influence our patterns significantly.

Finally, while it is interesting to speculate about the behaviour of colloidal liquid crystals in true 3D confinement, there are at present serious practical obstacles to doing so. On the experimental side, creating such cavities with the current state-of-the-art soft lithography techniques employed is unfortunately not possible. Although there are novel techniques on the horizon that could make fully 3D shapes realizable in glass sometime in the future^39^, such systems would likely face serious problems with proper filling. At the same time, a systematic simulation study of 3D containers is unfeasible with the computational resources currently at hand. To put this in perspective, single runs of some of the larger systems presented in this study require on the order of a few weeks of computer time on up-to-date hardware to equilibrate.

## Methods

### Confining geometry

The tactoid-like domains we employ are defined by the cross section of two overlapping circles of equal radius. The boundary is set by the arcs of the overlapping region, which gives an elongated lens-shaped domain with two cusps diametrically opposed with an opening angle *α*. The major axis of the spindle *L*_*y*_ is set by the distance between the vertices, while the minor axis *L*_*x*_ is the distances between the centers of the arcs, see Fig. 1(a). *α* represents the opening angle of the spindle-shaped confinement, also referred to as “angle of the wedge”. This angle can be related to the aspect ratio *L*_*y*_/*L*_*x*_ through *α* = 4 *arccot*(*L*_*y*_/*L*_*x*_).

### Experimental setup

Microchamber preparation was adapted from^15^. We designed a mask where we varied the major axis of the spindles between 10–70 *μ*m and the angle *α* between *α* = 180° (*L*_*y*_/*L*_*x*_ = 1 and therefore a circle) and *α* = 30° (*L*_*y*_/*L*_*x*_ = 7.59) in steps of 10°. Microchambers were designed with a major axis of 10 *μ*m, 20 *μ*m, 30 *μ*m, 50 *μ*m, and 70 *μ*m, respectively. Series of microchambers were vertically placed in a matrix, in the order of the major axis length. In each series, two rows of microchambers were arranged. The corresponding aspect ratio decreases accordingly from left to right, and from top to down (see Fig. 1(b)).

Shallow lens-shaped microchambers with a height of 3 *μ*m made of negative photoresist (SU-8, 2005, MicroChem) were prepared on top of clean cover slips by photolithography following a published procedure^15^. Briefly, a layer of SU-8 was spin-coated on the coverslips and baked twice (first at 75 °C for 15 min and next at 95 °C for 5 min). The sample was illuminated with UV irradiation through a custom-designed mask printed on soda-lime glas (DeltaMask) to define the chambers. Next the sample was baked for another 2 hours at 150 °C. Prior to experiments, the microchambers were passivated by immersion in sample buffer supplemented with 0.1 wt% of the amphiphilic block copolymer Pluronic F-127 (Sigma-Aldrich).

We used the rod-like bacteriophage *fd*-virus as our model liquid crystal system. *Fd*-virus rods are monodisperse and relatively stiff and slender, with a persistence length of 22 *μ*m and contour length of 880 nm and a diameter of 6.6 nm. The particle length is in the order of the size of the chambers and the particles are large enough to be imaged directly at the single particle level. The *fd*-virus rods were prepared following a published procedure^15,40^ and suspended in a 20 mM Tris buffer of pH 8.15, containing 100 mM sodium chloride, and 15% ethanol. We used the *fd*-virus rods at a final concentration *C*_*fd*_ = 24 mg/ml, which was chosen because it is close to the nematic-isotropic binodal but still in the fully nematic phase. A small fraction (1 in 1000) of the rods were fluorescently labeled with Alexa-488 succinimidyl ester (Invitrogen). The microchambers were filled by pipetting a drop of the *fd*-virus dispersion and sealed with a glass cover slip coated with a very thin layer of polydimethylsiloxane (PDMS).

Time-lapse images of the *fd*-virus samples in microchambers were recorded by a Nikon microscope equipped with a C1 confocal point scanner^41,42^. We took time-lapse movies of 30 frames at a rate of 1 frame per 1–2 minutes.

### Image analysis

We used a frame by frame image analysis method. The local alignment is determined using the plug-in “OrientationJ” of “ImageJ”. To the orientation image a threshold obtained from the brightness was applied. The orientation obtained in this way is averaged, taking into account the invariance of the system, over all the frames. The final images that is presented in Fig. 2 are colored according to the orientation and each pixel has a brightness obtained by the overlaying of the images. A more detailed description of the image analysis method can be found in the Supplementary Information of^15^.

### Simulation details

The confining geometry we used in simulations is the same as the experimental wells: the basis of the container is lens shaped (spindle-like), being fully characterized by the minor and major axis (*L*_*x*_, respectively *L*_*y*_), and the depth of the container *h* is much smaller than the length of either of the symmetry axes of the basis. We thus obtain a quasi- 2-dimensional volume, which we prefer over a strictly 2-dimensional system since this could lead to unphysical patterns due to jamming of particles (note that we are interested in a regime where the particle length is comparable with the confining volume). We have employed a standard Monte Carlo technique, modeling the *fd*-viruses as hard rods (spherocylinders with length *L* and diameter *d*) which interact with each other only through steric repulsion. The particle-wall interaction is also hard. We have considered confining containers with aspect ratio *L*_*y*_/*L*_*x*_ starting from 1 up to 5, *h*/*d* = 6 and values of *L*_*y*_/*L* in the interval [4.5, 20]. The rods length in our simulations is *L*/*d* = 20 and the volume fraction of the rods in the box is *η* = 0.16. Typical numbers of rods vary between hundreds and thousands of spherocylinders. The aspect ratio of the rods considered in simulations is considerably lower than the aspect ratio of the *fd*-viruses. However, this is not an impediment since we are able to scale down the system by taking into account that the length scale of the rod-wall interaction is in the order of one rod length. We therefore only need the ratio between the confinement size and the length of the rod to be of the same magnitude in simulations as it is in the experiments, regardless of the absolute lengths. The fact that our containers are so shallow that particles cannot freely rotate out of the basal plane, whereas in the experimental system they are in principle able to do so, is a less severe approximation than might be expected at first sight. In fact, as we have already discussed in^15^, the planar anchoring of particles to the large top- and bottom boundaries is so strong and has a penetration depth of at least the length of the particles, even the experimental system is effectively 2D, and therefore well represented by the computationally much more tractable shallow systems considered here.

### Defect characterization

Making the use of the standard tensor order parameter^43^, which reads:

1 $${\rm{Q}}=\frac{1}{N}\langle {\sum }_{i}(\frac{3}{2}{\hat{{\bf{u}}}}_{i}\otimes {\hat{{\bf{u}}}}_{i}-\frac{1}{2}{{\mathbb{1}}}_{3})\rangle ,$$

where ***u***_***i***_ is the orientation of rod *i*, it is only possible to gain information about the overall spatially averaged organization of the rods. The highest eigenvalue of this tensor corresponds to the degree of order in the system whereas the corresponding eigenvector gives the nematic director. We use these two quantities to determine the equilibration time. We have opted for the 3-dimensional version of this tensor so that we can also monitor the out of plane component.

To characterize the patterns observed in simulations we construct a local version of the the tensor order parameter. We achieve this by dividing the simulation box into small cuboidal sub-volumes of the same height *h*. For one such sub-volume (denoted *k*), the tensor order parameter is written as:

2 $${{\bf{Q}}}^{k}=\frac{1}{{\sum }_{i}{l}_{i}^{k}}\langle {\sum }_{i}{l}_{i}^{k}(\frac{3}{2}{\hat{{\bf{u}}}}_{i}\otimes {\hat{{\bf{u}}}}_{i}-\frac{1}{2}{{\mathbb{1}}}_{3})\rangle ,$$

where *l*_*i*_^*k*^ is the length of rod *i* that is located inside the sub-volume *k*. From the nematic director in the sub-volumes we can define an angular deficit parameter as follows:

3 $$\delta =min\angle ({\hat{{\bf{n}}}}^{1},{\hat{{\bf{n}}}}^{2})+min\angle ({\hat{{\bf{n}}}}^{3},{\hat{{\bf{n}}}}^{4}),$$

with ***n***^***j***^ orientations of neighboring sub-volumes. This parameter will vanish for a perfect nematic arrangement and will take singular positive values in the neighborhood of defects. For more details about the defect characterization we refer the reader to reference^35^ and the Supplementary Information of^15^.

## Supplementary information

Supplementary Information.

Supplementary Video 1.

Supplementary Video 2.

Supplementary Video 3.

## Data Availability

The datasets generated during and/or analysed during the current study are available from the corresponding author on reasonable request.
