# Supplementary material for: Colloidal Liquid Crystals Confined to Synthetic Tactoids
Source: Sci Rep. 2019 Dec 31;9:20391. doi: 10.1038/s41598-019-56729-9 (PMC6938498; doi:10.1038/s41598-019-56729-9)
Supplement: Supplementary file 1 — Supplementary Information. [file 41598_2019_56729_MOESM1_ESM.doc]

Supplementary Information

Colloidal Liquid Crystals Confined to Synthetic Tactoids

*Ioana C. Gârlea, Oliver Dammone, José Alvarado, Valerie Notenboom, Yunfei Jia, Gijsje H. Koenderink, Dirk G. A. L. Aarts, M. Paul Lettinga, and Bela M. Mulder*

Captions Supplementary Movies:

Supplementary Movie S1: Equilibration trajectory for the “melt” pathway. The color of the rods changes from light blue, for rods aligned with the *L_y_* axis, to yellow, for rods oriented at a 45° angle with respect to the axis, to magenta for rods aligned with the *L_x_*. One of the rods has been represented with a different color code (changing continuously from red for horizontally oriented rods (along *L_x_*) to yellow for rods along the L_y_ direction) and with twice the diameter of the other rods to make it easily trackable. Simulation parameters: *L_y_/L_x_* =1.5 and *L_y_/L*=6.

Supplementary Movie S2: Equilibration trajectory for the “slide” pathway. Color code of the particles is the same as for the Supporting Movie 1. Simulation parameters: *L_y_/L_x_* =1.5 and *L_y_/L*=10.5.

Supplementary Movie S3: Equilibration trajectory for the “turn” pathway. Color code of the particles is the same as for the Supporting Movie 1. Simulation parameters: *L_y_/L_x_* =1.5 and *L_y_/L*=15.
